# Supplementary material for: Uncovering population contributions to the extracellular potential in the mouse visual system using Laminar Population Analysis
Source: bioRxiv. 2024 Jan 16:2024.01.15.575805. Preprint. [Version 1] doi: 10.1101/2024.01.15.575805 (PMC10827114; doi:10.1101/2024.01.15.575805)
Supplement: Supplement 1 [file NIHPP2024.01.15.575805v1-supplement-1.pdf]

bioRxiv preprint doi: https://doi.org/10.1101/2024.01.15.575805; this version posted January 16, 2024. The copyright holder for this preprint (which was not certified by peer review) is the author/funder, who has granted bioRxiv a license to display the preprint in perpetuity. It is made available under aCC-BY 4.0 International license.

Supporting information

Supplementary Figures

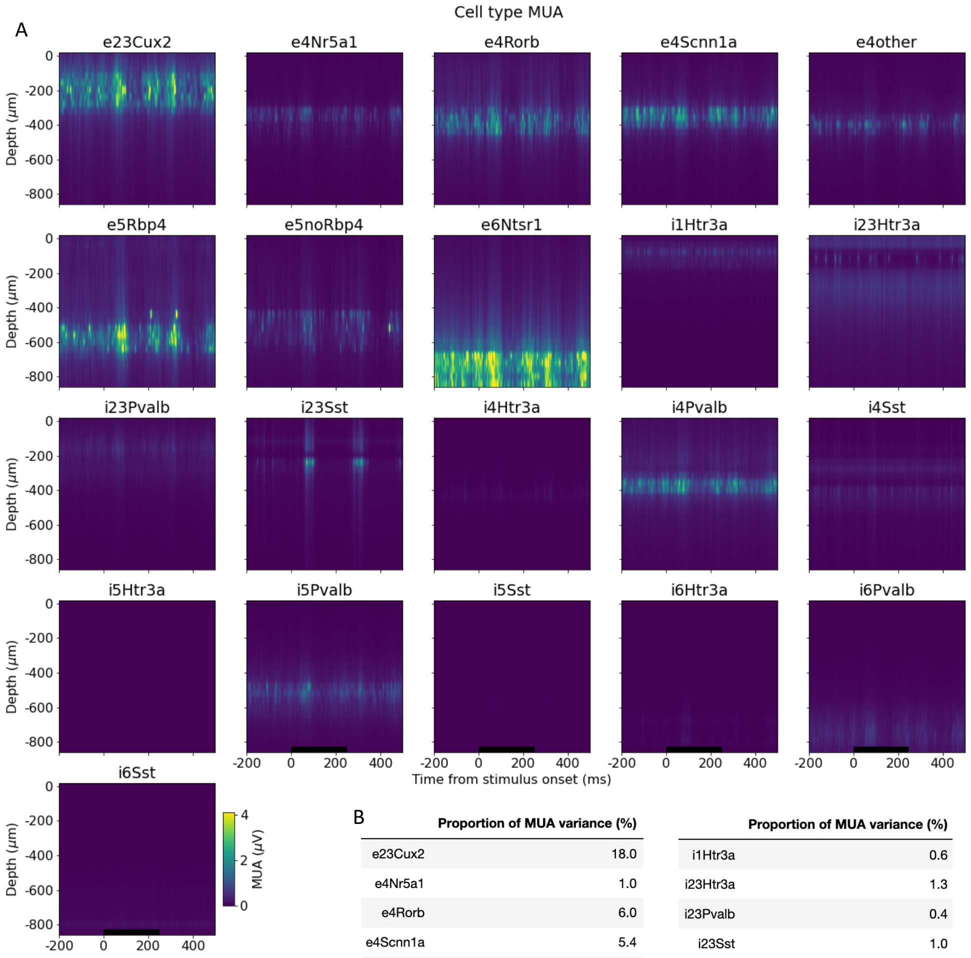

B

	Proportion of MUA variance (%)
e23Cux2	18.0
e4Nr5a1	1.0
e4Rorb	6.0
e4Scnn1a	5.4
e4other	1.5
e5Rbp4	17.4
e5noRbp4	3.0
e6Ntsr1	36.3
Sum	88.5

	Proportion of MUA variance (%)
i1Htr3a	0.6
i23Htr3a	1.3
i23Pvalb	0.4
i23Sst	1.0
i4Htr3a	0.4
i4Pvalb	3.9
i4Sst	0.4
i5Htr3a	0.4
i5Pvalb	1.4
i5Sst	0.4
i6Htr3a	0.4
i6Pvalb	0.5
i6Sst	0.3
Sum	11.5

Fig. S1. MUA variance by cell types. (A) MUA contribution of cell type families in the model. (B) Proportion of variance explained by excitatory (left) and inhibitory (right) cell type families.

bioRxiv preprint doi: https://doi.org/10.1101/2024.01.15.575805; this version posted January 16, 2024. The copyright holder for this preprint (which was not certified by peer review) is the author/funder, who has granted bioRxiv a license to display the preprint in perpetuity. It is made available under aCC-BY 4.0 International license.

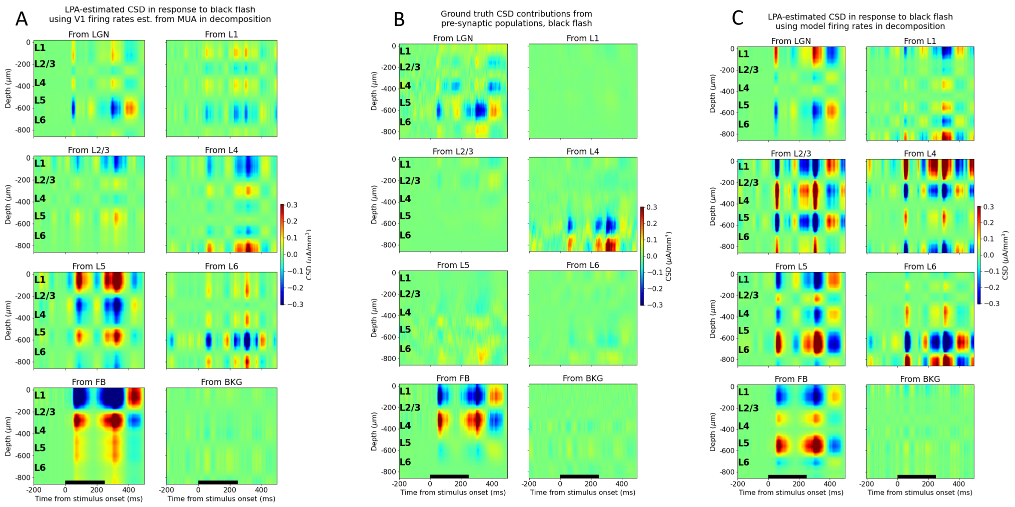

	Relative MSE	Correlation
LGN	0.5	0.73
L1	86.8	-0.20
L2/3	11.6	0.01
L4	1.0	0.47
L5	19.5	-0.06
L6	17.2	0.47
FB	1.4	0.79
BKG	1.2	0.51

	Relative MSE	Correlation
LGN	1.2	0.47
L1	183.3	0.45
L2/3	184.5	-0.27
L4	7.4	-0.16
L5	21.2	0.06
L6	62.1	0.48
FB	1.0	0.53
BKG	2.3	0.28

Fig. S2 Black flash estimates from LPA on simulated CSD with V1 laminar populations distinguished. (A) Top: Black flash CSD generated from firing in each presynaptic population estimated with LPA using temporal profiles obtained from MUA as shown in fig. 2D. Bottom: Relative MSE and correlation between LPA-estimated and true black flash CSD contributions from each presynaptic population. (B) True Black flash CSD contributions from firing in each presynaptic population. (C) Top: Black flash CSD generated from firing in each presynaptic population estimated with LPA using temporal profiles obtained from firing rates of excitatory cells in the model in each layer from 2/3 to 6 and inhibitory cells in layer 1. Bottom: Relative MSE and correlation between LPA-estimated and true black flash CSD contributions from each presynaptic population.

bioRxiv preprint doi: https://doi.org/10.1101/2024.01.15.575805; this version posted January 16, 2024. The copyright holder for this preprint (which was not certified by peer review) is the author/funder, who has granted bioRxiv a license to display the preprint in perpetuity. It is made available under aCC-BY 4.0 International license.

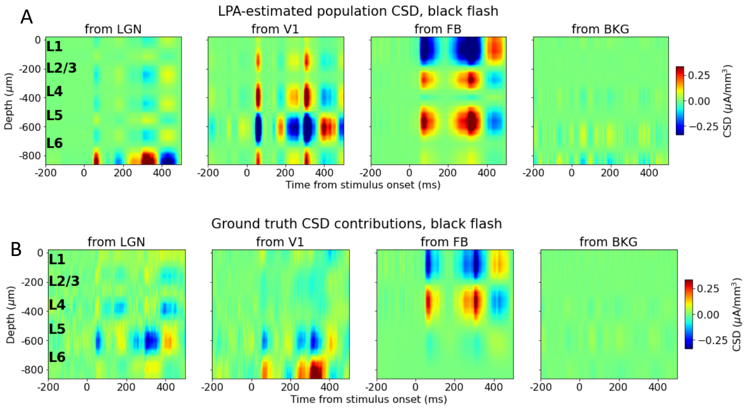

C

	Relative MSE	Correlation
LGN	1.9	0.20
V1	3.1	0.45
FB	2.1	0.61
BKG	4.6	0.50

Fig. S3. Black flash estimates from LPA on simulated CSD with one population for V1. (A) Top: LPA-estimated black flash CSD generated from firing in each presynaptic population. Firing rates of excitatory cells in the model are used for the presynaptic temporal profiles of V1 in the decomposition. (B) True black flash CSD generated from firing in the same presynaptic populations. (C) Relative MSE and correlation between LPA-estimated and true black flash CSD generated from each presynaptic population.

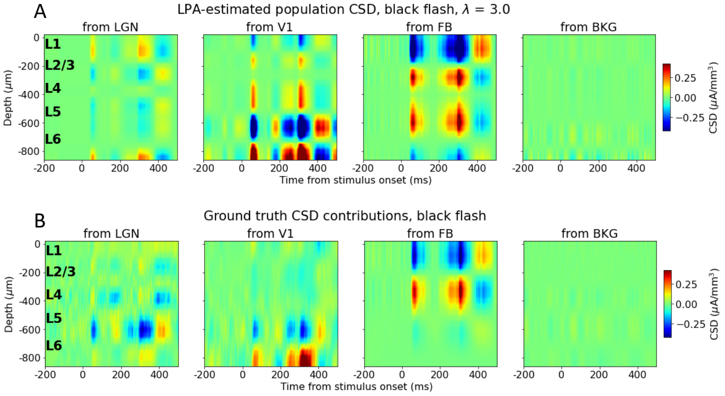

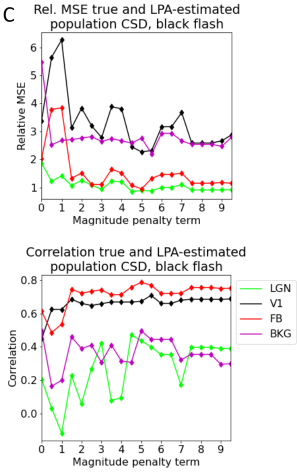

D

	Relative MSE	Correlation
LGN	0.9	0.42
V1	2.8	0.66
FB	1.1	0.74
BKG	2.6	0.31

Fig. S4. Black flash estimates from LPA on simulated CSD with penalty on deviations from zero in CSD summed across channels. (A) LPA-estimated black flash CSD generated from firing in each presynaptic population for $\lambda = 3$, the value at which the deviation from 0 in the CSD summed across channels was smallest (Fig. 5A). (B) True black flash CSD generated from firing in presynaptic populations. (C) Relative MSE (left) and correlation (right) between CSD generated from each presynaptic population estimated by CSD and true CSD generated from each presynaptic population at different values of λ. LGN: green, V1: black, feedback: red, background: purple. (D) Relative MSE and correlation between LPA-estimated and true black flash CSD generated from each presynaptic population at $\lambda = 3$.

bioRxiv preprint doi: https://doi.org/10.1101/2024.01.15.575805; this version posted January 16, 2024. The copyright holder for this preprint (which was not certified by peer review) is the author/funder, who has granted bioRxiv a license to display the preprint in perpetuity. It is made available under aCC-BY 4.0 International license.

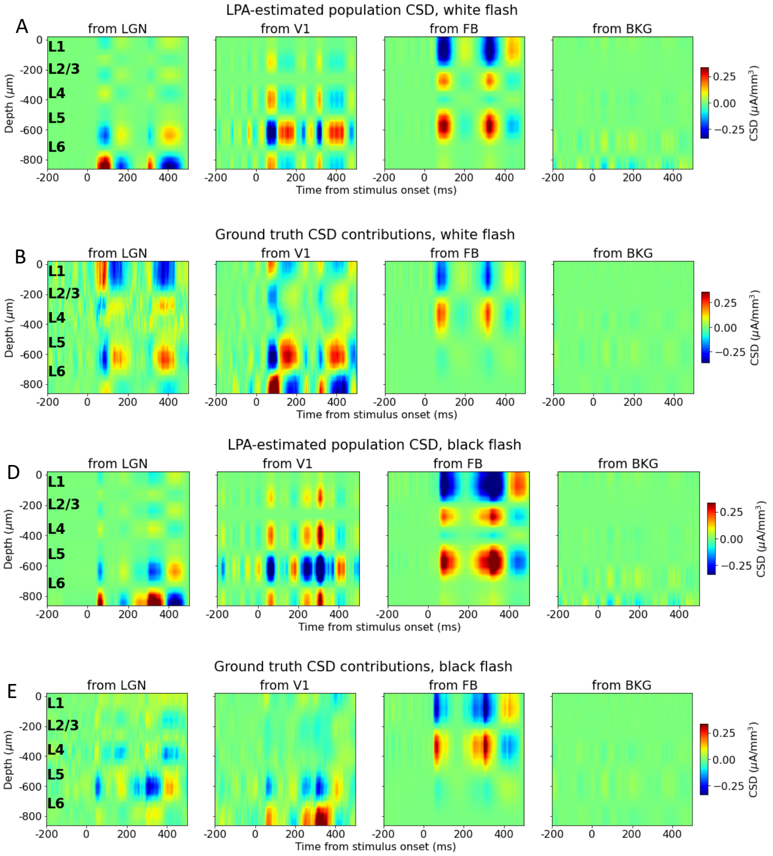

C

	Relative MSE	Correlation
LGN	1.1	0.31
V1	0.6	0.61
FB	1.9	0.54
BKG	3.4	0.36

F

	Relative MSE	Correlation
LGN	1.3	0.52
V1	2.0	0.50
FB	2.3	0.54
BKG	3.4	0.36

Fig. S5. LPA on simulated CSD using temporal profiles estimated from MUA in decomposition and one population for V1. (A) Top: LPA-estimated white flash CSD generated from firing in each presynaptic population. (B) True white flash CSD generated from firing in the same presynaptic populations. (C) Relative MSE and correlation between LPA-estimated and true white flash CSD generated from each presynaptic population. (D) Top: LPA-estimated black flash CSD generated from firing in each presynaptic population. (E) True black flash CSD generated from firing in the same presynaptic populations. (F) Relative MSE and correlation between LPA-estimated and true black flash CSD generated from each presynaptic population.

bioRxiv preprint doi: https://doi.org/10.1101/2024.01.15.575805; this version posted January 16, 2024. The copyright holder for this preprint (which was not certified by peer review) is the author/funder, who has granted bioRxiv a license to display the preprint in perpetuity. It is made available under aCC-BY 4.0 International license.

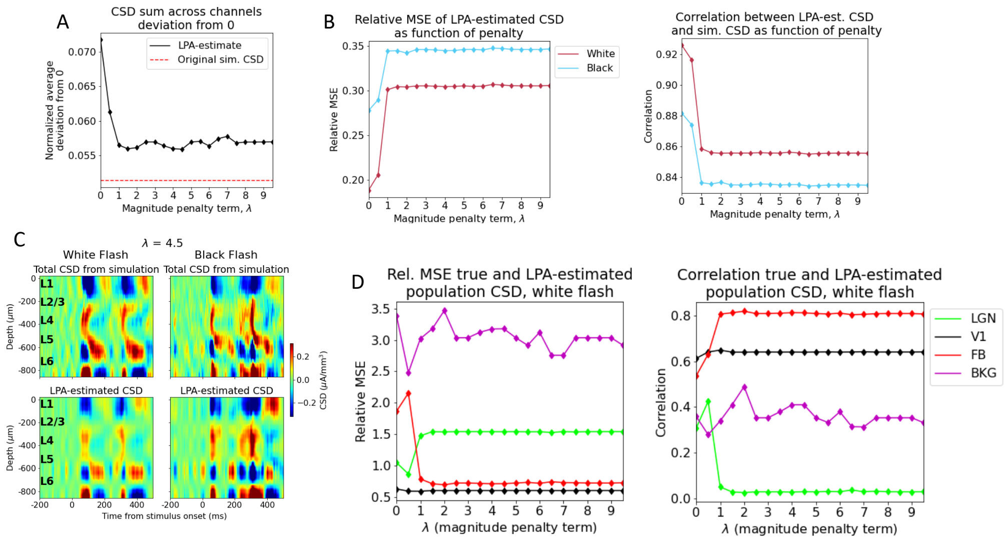

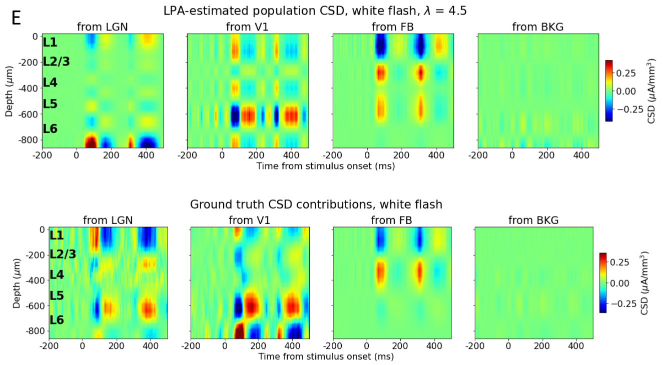

F

	Relative MSE	Correlation
LGN	1.5	0.03
V1	0.6	0.64
FB	0.7	0.81
BKG	3.2	0.41

Fig. S6. Penalizing deviations from zero in CSD summed across channels with presynaptic temporal profiles estimated from MUA. (A) Average sum of LPA-estimated total CSD across channels (black line) with varying penalty (λ) on deviations from 0. Red dashed line: Average deviation from 0 of sum across channels for total simulated CSD to which LPA was applied. (B) Relative MSE (left) and correlation (right) between CSD from simulation and CSD estimated from LPA-components at different penalty magnitudes with temporal profile of V1 firing rates estimated from MUA. White flash: dark red line, black flash: blue line. (C) Total simulated CSD (top) and CSD estimated from LPA-components (bottom) in response to white and black full-field flash when the penalty for deviations from zero $\lambda = 4.5$, which is the value at which the deviation from zero for the average sum across channels in the LPA-estimated CSD is smallest (see (A)). (D) Relative MSE (left) and correlation (right) between LPA-estimated and true white flash CSD generated from each presynaptic population at different values of λ. LGN: green, V1: black, feedback: red, background: purple. (E) Top: LPA-estimated white flash CSD generated from firing in each presynaptic population for $\lambda = 4.5$. Bottom: True white flash CSD generated from firing in presynaptic populations. (F) Relative MSE and correlation between LPA-estimated and true white flash CSD generated from each presynaptic population with $\lambda = 4.5$.

bioRxiv preprint doi: https://doi.org/10.1101/2024.01.15.575805; this version posted January 16, 2024. The copyright holder for this preprint (which was not certified by peer review) is the author/funder, who has granted bioRxiv a license to display the preprint in perpetuity. It is made available under aCC-BY 4.0 International license.

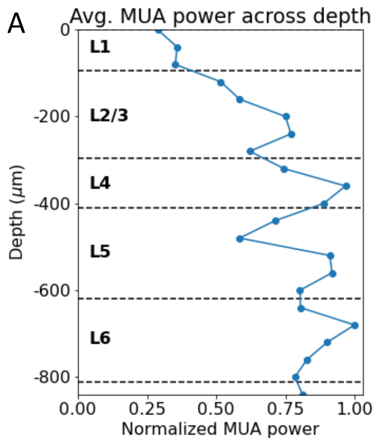

Fig. S7. Avg. MUA power across depth in V1 model. (A) The blue line shows the average MUA power at different channels of the simulated probe across all trials of white and black full-field flash stimuli.

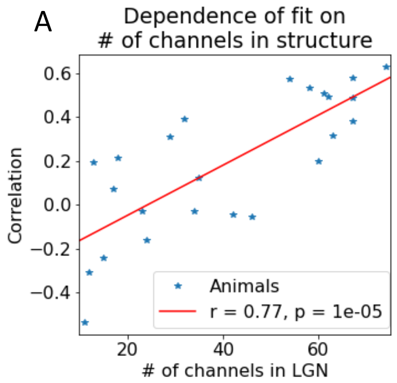

Fig. S8. MUA fit depends on number of recording channels in LGN. (A) Relationship between number of recording channels in LGN in experiments and the correlation between the LPA-estimated and simulated MUA in LGN. Each blue star corresponds to an animal in the dataset and the red line displays the results of a linear regression between number of recording channels in LGN and the correlation.

bioRxiv preprint doi: https://doi.org/10.1101/2024.01.15.575805; this version posted January 16, 2024. The copyright holder for this preprint (which was not certified by peer review) is the author/funder, who has granted bioRxiv a license to display the preprint in perpetuity. It is made available under aCC-BY 4.0 International license.

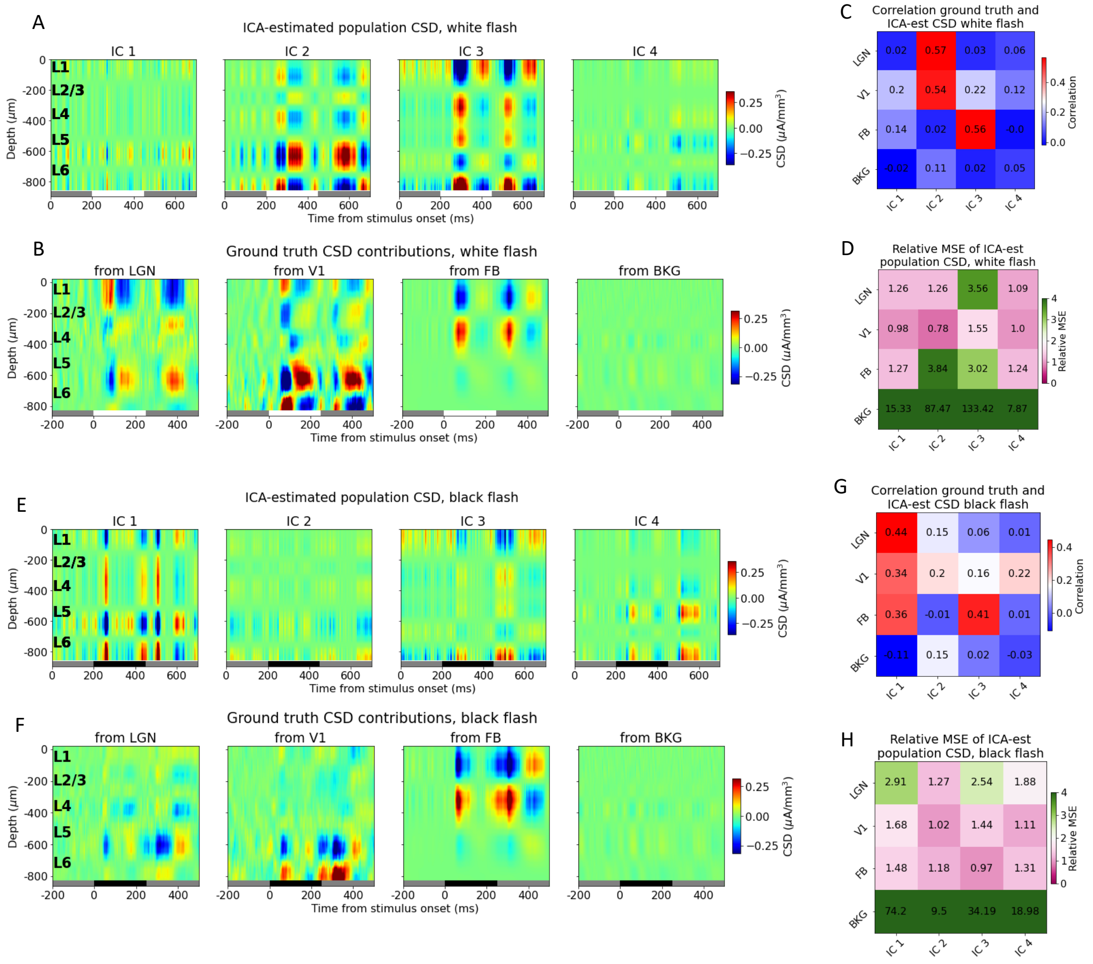

Fig. S9. ICA cannot identify the four major CSD contributors. (A) Estimated white flash CSD of four ICA components. (B) True white flash CSD from each population. (C-D) Correlation and relative MSE, respectively, between CSD of ICA-components and true CSD in (A) and (B). (E) Estimated black flash CSD of four ICA components. (F) True black flash CSD from each population. (G-H) Correlation and relative MSE, respectively, between CSD of ICA-components and true CSD in (E) and (F).

bioRxiv preprint doi: https://doi.org/10.1101/2024.01.15.575805; this version posted January 16, 2024. The copyright holder for this preprint (which was not certified by peer review) is the author/funder, who has granted bioRxiv a license to display the preprint in perpetuity. It is made available under aCC-BY 4.0 International license.

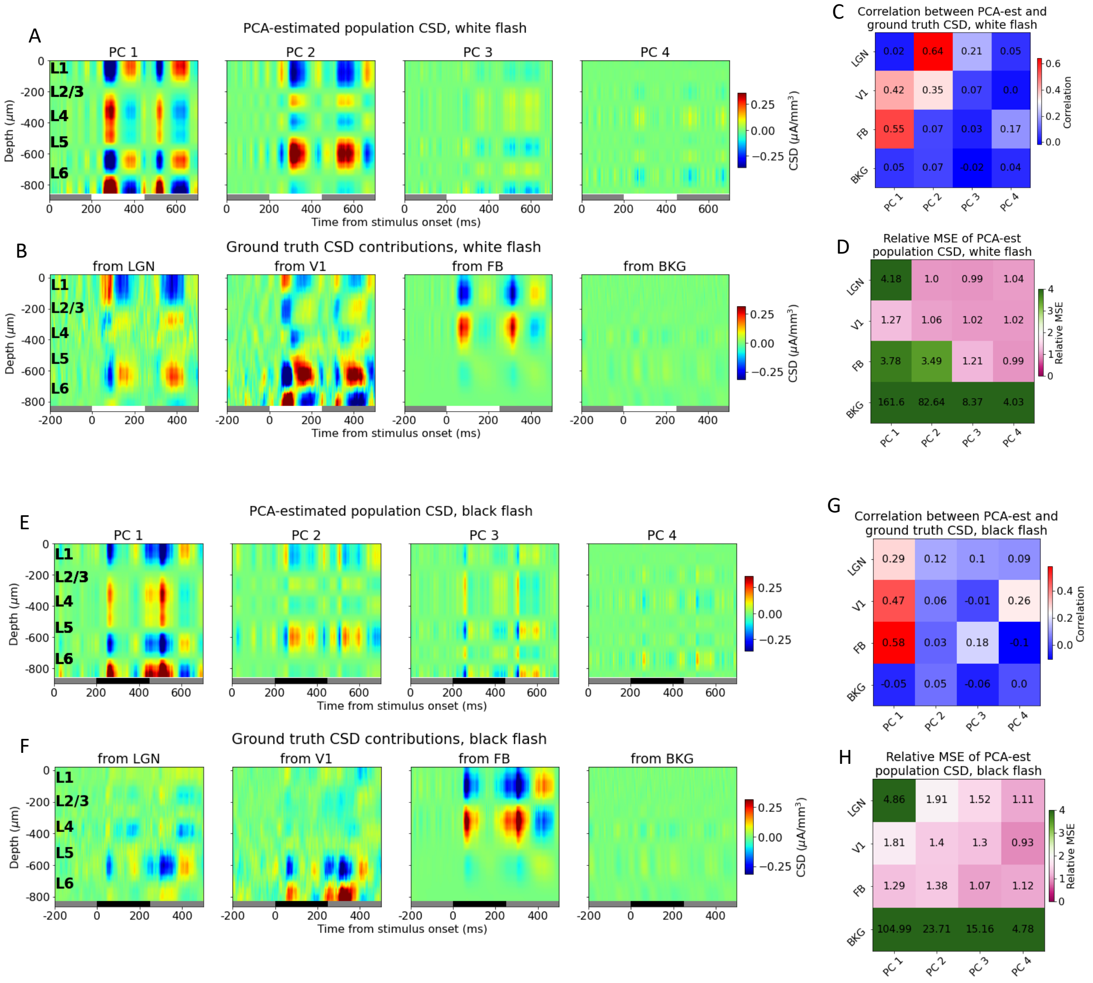

Fig. S10. PCA cannot identify the four major CSD contributors. (A) Estimated white flash CSD of four PCA components. (B) True white flash CSD from each population. (C-D) Correlation and relative MSE, respectively, between CSD of PCA-components and true CSD in (A) and (B). (E) Estimated black flash CSD of four PCA components. (F) True black flash CSD from each population. (G-H) Correlation and relative MSE, respectively, between CSD of ICA-components and true CSD in (E) and (F).

bioRxiv preprint doi: https://doi.org/10.1101/2024.01.15.575805; this version posted January 16, 2024. The copyright holder for this preprint (which was not certified by peer review) is the author/funder, who has granted bioRxiv a license to display the preprint in perpetuity. It is made available under aCC-BY 4.0 International license.

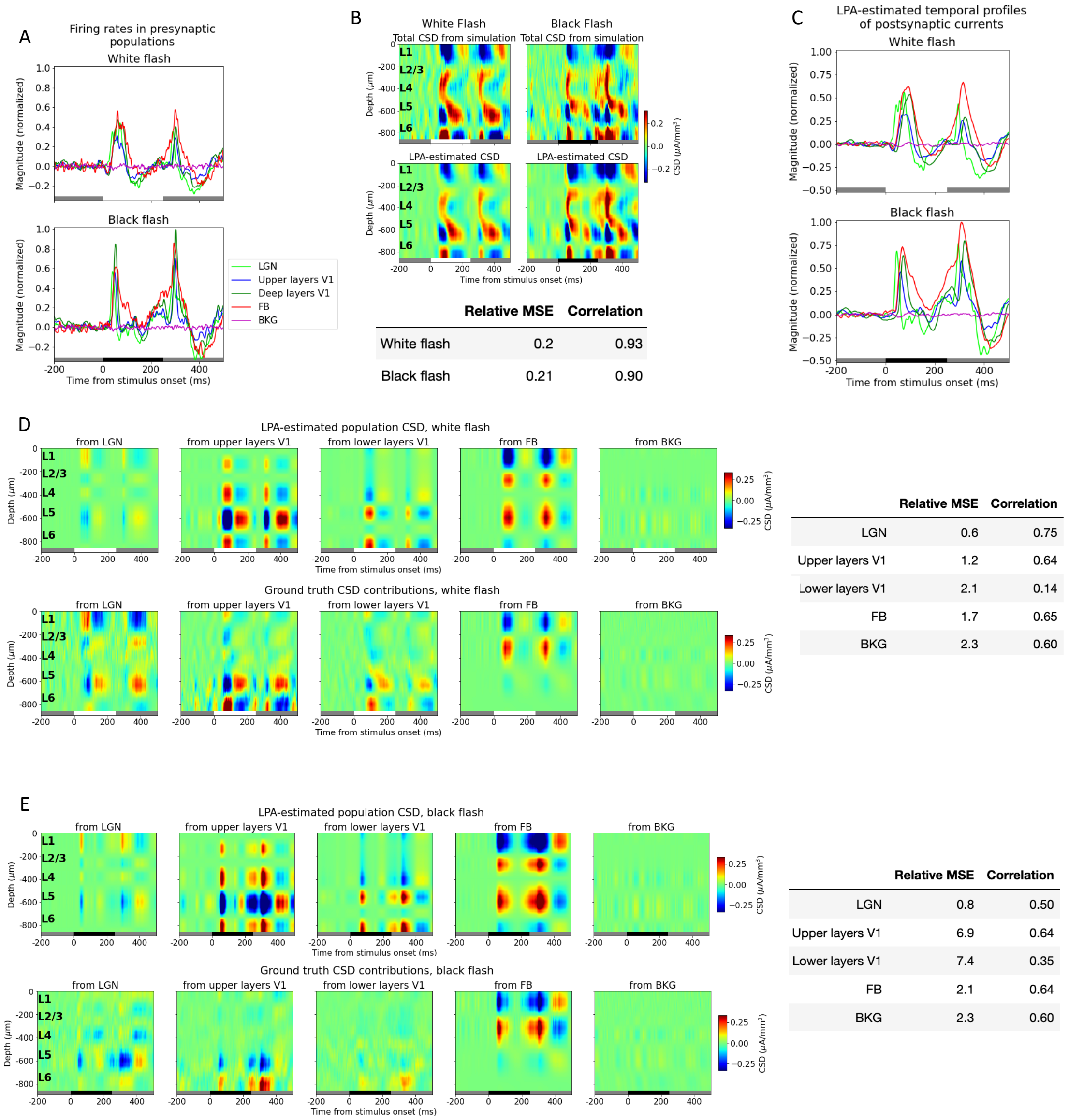

Fig. S11. Applying LPA to simulated data with two populations for V1 distinguished. (A) Temporal profiles of firing rates of presynaptic excitatory populations in external structures (LGN: light green line; feedback: red line; background: purple line), upper layers V1 merged (blue line), and deep layers V1 merged (dark green line). (B) Top: Trial-averaged CSD from simulation with white (left) and (black) flash stimuli and CSD estimated from LPA-components using presynaptic firing rates in (A) in decomposition. Bottom: Relative MSE and correlation between CSD from simulation and CSD estimated from LPA-components. (C) Temporal profiles of CSD of each LPA-component in response to white and black flash. (D) Top: LPA-estimated white flash CSD generated from firing in each presynaptic population shown in (A). Bottom: True white flash CSD generated from firing in the same presynaptic populations. Right: Relative MSE and correlation between LPA-estimated and true white flash population CSD. (E) Top: LPA-estimated black flash CSD generated from firing in each presynaptic population shown in (A). Bottom: True black flash CSD generated from firing in the same presynaptic populations. Right: Relative MSE and correlation between LPA-estimated and true black flash population CSD.
